# Current Trends and Biotechnological Innovations in Biofouling Control of RO Membranes in Desalination Systems

**DOI:** 10.3390/membranes15090270

**Published:** 2025-09-05

**Authors:** Victoria Cruz-Balladares, Hernán Vera-Villalobos, Carlos Riquelme, Fernando Silva Aciares

**Affiliations:** 1 Centro de Bioinnovación de Antofagasta, Facultad de Ciencias del Mar y Recursos Biológicos, Universidad de Antofagasta, Antofagasta 1240000, Chile; victoria.cruz@uantof.cl (V.C.-B.); hernan.vera@uantof.cl (H.V.-V.); carlos.riquelme@uantof.cl (C.R.); 2 Departamento de Biotecnología, Facultad de Ciencias del Mar y Recursos Biológicos, Universidad de Antofagasta, Antofagasta 1240000, Chile

**Keywords:** biofouling, antifouling, reverse osmosis, biosurfactants, quorum-quenching, desalination

## Abstract

Background: Water scarcity is a pressing global challenge increasingly addressed by advanced desalination that converts seawater into potable water. Reverse osmosis and ultrafiltration dominate because they deliver permeate with very low impurities. Their principal limitation is membrane biofouling, which causes clogging, raises energy, operation, and maintenance costs, and shortens membrane life. Multiple approaches mitigate biofouling—most notably pretreatment trains and engineered surface coatings—but cleaning remains the most decisive remediation pathway. Current practice distinguishes physical, chemical, and biological cleaning. Biological cleaning has gained momentum by exploiting microorganisms that inherently counter biofilms. These strategies include targeted secretion of enzymes and antifouling metabolites, and the application of whole-cell culture supernatants containing the full suite of secreted components. In addition, predatory bacteria can infiltrate established biofilms and eradicate them by lysing prey, thereby accelerating the removal of adherent biomass. Progress across these bio-based approaches signals meaningful advances in fouling control and could substantially improve the efficiency, reliability, and sustainability of desalination facilities. Collectively, they underscore the transformative potential of biological antifouling agents in operational systems. Realizing that potential will require rigorous evaluation of technical performance, long-term stability, compatibility with polyamide membranes, regulatory acceptance, and environmental safety, ultimately alongside scalable production and cost-effective deployment in full-scale plants.

## 1. Introduction

The global population is projected to increase to 9.7 billion by 2050 [1]. Consequently, addressing water scarcity has emerged as a critical global challenge in recent times, necessitating the exploration of methods to provide an adequate supply of potable water [2]. This issue is exacerbated by factors such as industrialization, pollution, and climate change. In response, the implementation of desalination technologies has gained prominence, particularly in coastal regions, where seawater can be harnessed as a valuable resource for both industrial applications, including energy generation and mining, and for the provision of drinking water [3]. The International Desalination Association (IDA) reports that over 150 countries have adopted desalination as a means of freshwater supply. Multiple sector reviews document broad global adoption of desalination [4,5]. In parallel, coupling reverse osmosis desalination with renewable energy (e.g., wind power) has been comprehensively reviewed as a clean power pathway [6,7].

To frame subsequent biotechnological approaches, we first summarize state-of-practice non-biological controls placed upstream of, or applied periodically to, the following RO elements: (i) pretreatment trains (e.g., coagulation–flocculation, filtration, UVC/ozone) that reduce biofoulant load [8,9,10,11,12]; (ii) chemical/physical clean-in-place (CIP) protocols that restore flux using alkali/acid oxidants and hydraulics [13,14]; and (iii) membrane surface modification/coatings that impart anti-adhesive or biocidal characteristics [15,16,17,18]. This addition clarifies the baseline against which biological innovations are compared and avoids conflating preventive with restorative measures [19].

In the 1960s, the initial desalination plants were established in the Middle East, with Saudi Arabia taking a leading role by producing 22% of the world’s desalinated water. In 2004, the first reverse osmosis desalination plants in South America commenced operations in Antofagasta, northern Chile, to support the Minera Escondida Limitada sulfide leaching project [20].

Numerous water desalination methodologies are utilized to derive potable water from coastal regions, which can be broadly categorized into two main groups: thermal distillation and evaporation processes, and those employing physical membranes as the principal purification mechanism, including electrodialysis, membrane distillation [21], and reverse osmosis. Notably, despite the array of available desalination technologies, reverse osmosis membranes predominate as the core desalination step [3], whereas ultrafiltration systems are used extensively as pretreatment to protect RO from particulate and biological fouling. UF does not itself desalinate seawater due to its effectiveness in large-scale applications, particularly concerning cost-to-production ratios for potable water [22]. The utilization of reverse osmosis offers significant advantages, as it provides satisfactory water permeability alongside high rejection rates for organic, inorganic, and pathogenic micropollutants. Conversely, a notable drawback is that due to fouling, reverse osmosis membranes typically have a lifespan of 5 to 10 years; after this period, most membranes are disposed of in landfills. However, alternatives such as reuse, where membranes are repurposed for applications requiring lower water quality, and recycling, which involves chemical modification to restore functionality, exist. Consequently, the optimization of these processes constitutes a crucial aspect of ensuring long-term sustainability, enhancing efficiency, and mitigating potential ecological impacts [23,24,25,26].

One of the most significant challenges in reverse osmosis desalination systems is the emergence of biofouling on membranes, which has implications for operational and maintenance costs [27,28]. Comparable challenges are documented in marine aquaculture, where biofouling severely impacts net pens and farm infrastructure in contact with both fresh and saline water [29]. This is particularly pertinent to desalination plant systems, notably ultrafiltration systems (UF) and reverse osmosis membranes (ROMs). Consequently, this leads to a progressive decline in the permeate rate, an increase in solute passage over time, and adverse effects on system performance, which subsequently result in downtime associated with maintenance and cleaning, as well as the eventual need for membrane replacement, thereby incurring additional costs [30]. This review aims to critically synthesize current biological and biotechnological approaches to fouling mitigation, with a focus on microbial solutions applicable to RO desalination systems.

**Figure 1:**
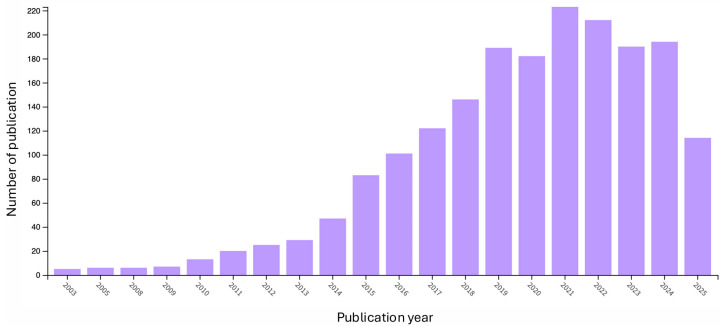
Number of publications related to the mitigation of membrane fouling in reverse osmosis systems (retrieved from the Web of Science database, July 2025, using the keywords (desalination plant OR reverse osmosis) AND (mitigation OR antifouling).

From a regulatory perspective, the integration of biologically derived cleaners or live/engineered quorum-quenching agents into potable water trains must align with the World Health Organization (WHO) Guidelines for Drinking-water Quality (GDWQ) [31], the European Food Safety Authority (EFSA) Qualified Presumption of Safety (QPS) approach [32], the United States Environmental Protection Agency (US EPA) Toxic Substances Control Act (TSCA) biotechnology regulations (40 CFR Part 725) [33], and the European Union (EU) Water Framework Directive (WFD) [34,35]. Organotin-based antifouling systems are controlled under the IMO’s International Convention on the Control of Harmful AntiFouling Systems on Ships (adopted in 2001), which set a global ban on application from 1 January 2003 and required ships by 1 January 2008 either to be free of such compounds or to have a sealing barrier coat [35]. It is important to note that fouling impacts all membrane types employed in desalination processes, including direct osmosis, membrane distillation, reverse osmosis, and ultrafiltration systems. Regardless of the membrane utilized, there is a continuous pursuit of novel strategies for fouling mitigation. Consequently, there is an urgent need to identify new alternatives for membrane cleaning in order to extend their operational lifespan and ensure an adequate water supply for the population. This need has been evidenced by a sustained increase, over at least the past ten years, in research and scientific publications focused on fouling mitigation in desalination systems (Figure 1) [29,36].

The continuous application of membranes results in the persistent accumulation of fouling on the surface and/or internal pores of the membrane, leading to obstruction and a reduction in permeate flow [15]. Membrane fouling is classified into four categories: inorganic fouling, which is predominantly due to mineral salts such as Ca^2+^, Mg^2+^, Ba^2+^, Na^+^, CO_3_^2−^, SO_4_^2−^, Cl^−^, and PO_4_^3−^, as well as silicates and silica; organic fouling, characterized by the deposition of dissolved organic matter on the membrane surface, including low molecular weight humic substances, polysaccharides (such as alginate), and proteins; colloidal fouling, involving intermediate-sized particles that may be inorganic (salts), organic (proteins, polysaccharides, etc.), or biological (microorganisms), which are particularly challenging to eliminate due to their size relative to pretreatment systems and membrane surfaces; and biofouling, which is associated with organic fouling, as microorganisms such as bacteria, fungi, and algae proliferate on organic materials from the surrounding environment, adhering to surfaces and gradually forming biofilms. These microorganisms secrete extracellular polymers (EPS) that facilitate the formation of complex biofilms that are difficult to eradicate [12,15,16,37]. The removal of these biofilms necessitates the implementation of various membrane cleaning or treatment methods [37]. For an up-to-date synthesis on marine biofilm diversity, early colonizers and their role in macrofouling, see [38].

Biofouling, in its initial state known as microfouling, is characterized by an aggregation of microbial cells at an interface, which are encapsulated within an extracellular polymeric substance (EPS) matrix. Specifically, in seawater desalination plants, biofouling results in a decline in the efficacy of ultrafiltration modules and a reduction in permeability flux across reverse osmosis membranes. This phenomenon necessitates elevated pressure to facilitate water movement within these systems, from the feed inlet of a component to the outlet, thereby augmenting energy expenses [17,18].

## 2. Cleaning Reverse Osmosis Membrane Systems

To prolong the operational lifespan of reverse osmosis membranes utilized in the desalination industry, a range of preventive and corrective strategies has been employed. These strategies include water pretreatment technologies [20], membrane surface modification (coating) [22], and reverse osmosis membrane washing, addressing technical, chemical, physical, and biological dimensions. Each of these technologies possesses specific technical and economic merits and drawbacks, which may arise from factors such as implementation, maintenance, or waste disposal.

### 2.1. Antifouling Methods

Membrane washing strategies are employed when membrane performance diminishes by 10 to 15%, resulting in a decrease in permeability, necessitating cleaning interventions through the partial cessation of water production. This process involves the removal of membranes affected by biofouling and their subsequent introduction into washing modules. Various washing strategies are available to address fouling in desalination systems, which are classified based on their characteristics. It is important to note that none of these systems completely eradicates microfouling; however, they are effective in eliminating a portion of it, thus facilitating the restoration of permeate volumes and recovering a portion of production [22].

In this context, the four key criteria that a chemical treatment must fulfill to be deemed effective are delineated by Madaeni and Samieirad (2010) [13]. Specifically, these criteria include the following: (1). The ability to release and dissolve fouling-promoting agents; (2). The capacity to maintain these agents in solution; (3). The prevention or mitigation of new scaling; and (4). The avoidance of any adverse effects on the integrity of the membrane or other components of the desalination system.

The initial antifouling agents employed were chemical in nature, most notably tributyltin (TBT) during the 1950s. This highly toxic biocide elicited detrimental effects in marine organisms and humans, including genetic mutations, which ultimately led to its prohibition in 2008 by the International Maritime Organization (IMO) [35]. Over time, environmental regulations have constrained the use of certain compounds, thereby driving the development of alternative methodologies. One such methodology involves the utilization of gases, specifically nitric oxide (NO), which is effective in dispersing biofilms prior to cell inactivation [39]. Nevertheless, acidic and alkaline chemical agents are currently the most prevalent due to their cost-effectiveness and ease of application. These treatments, however, possess adverse effects, such as the reduction in the useful life of reverse osmosis membranes and the necessity for waste management facilities to mitigate environmental impacts. The imperative to develop alternative antifouling treatments with reduced side effects is underscored by the fact that chemical treatments can induce bacterial resistance, including chlorine tolerance, which exacerbates long-term risks (Table 1). Conversely, physical strategies have been proposed, such as the use of ultraviolet-C (UVC) doses at various wavelengths, demonstrating effective fouling control. Additionally, the combination of ozone and ultraviolet light under vacuum conditions has demonstrated efficacy in mitigating biofouling on membranes. Ultrasound technology has also been applied, producing microbubbles that generate turbulence, thereby interrupting membrane fouling and enhancing water flow [40]. Another approach involves the treatment of membranes at elevated temperatures, with exposure to temperatures reaching up to 80 °C, which induces cell lysis and disintegration of exopolymeric substances and colloidal organic compounds, thereby liberating the membranes from fouling. Although these strategies yield promising results in addressing fouling, their high costs warrant the exploration of antifouling alternatives. Emerging nanostructured coatings—such as graphene oxide, silver nanoparticles, and photocatalytic TiO_2_—are also under investigation for enhanced desalination performance and, in some cases, antifouling [41,42].

### 2.2. Biological Methods, Biofouling: Problem and Opportunity?

Beyond constraints, biofouling ecology presents actionable opportunities: (i) mining biofilm consortia for robust EPS-depolymerizing enzymes (alginate lyases, DNases, proteases) [49,50]; (ii) valorizing cell-free supernatants from marine bacteria as low-toxicity cleaning cocktails [51,52,53,54]; (iii) engineering bio-conditioned surfaces with transient, non-colonizing quorum sensing (QS) inhibitors to delay attachment [55,56]; and (iv) combining bio-inspired chemistries (e.g., (+)-terrein grafting) with thin-film composite design to deliver durable anti-adhesive function [57].

Biofouling represents a complex biological environment [38], characterized by numerous interactions among microorganisms that inhabit a shared physical space. Specifically, certain interactions can augment biofouling production through quorum-sensing molecules, such as N-acyl homoserine lactones (AHLs) or autoinducer peptides (AIPs), which promote extracellular polymeric substance (EPS) secretion [58]. These quorum-sensing-mediated processes—namely, autoinducer synthesis, signal release, and receptor-dependent transcription—occur alongside cooperation, synergy, cohesion, and co-aggregation events that establish the foundations for biofilm formation, ultimately impacting plant operations through process disruption, reduced permeate production, and economic losses. However, in conditions of resource scarcity, microbial competition becomes apparent [59]. Within this framework, various microbes, including bacteria, fungi, and phytoplankton, produce bioactive compounds that, in some instances, involve the synthesis of secondary metabolites capable of mediating competitive interactions. Notably, several substances that mediate bacterial competition are secreted molecules produced during the stationary phase, including enzymes (e.g., lipases, proteases, DNases, and other enzymes) and bioactive small peptides, which can be employed against the initial stages of biofilm formation, such as antimicrobial peptides (AMPs) [60] or bacteriocins [61,62]. For example, Santagati et al. (2012) [63] demonstrated that Streptococcus salivarius produces several bacteriocins that inhibit different Gram-positive pathogens in the upper respiratory tracts. *Lactobacillus* species can secrete several glycoproteins that prevent the attachment of potential pathogens in the gastrointestinal tract. *Pseudomonas fluorescens* produces chelator compounds that give an advantage over non-chelator bacteria in biofilms. Lactobacillus species can secrete several glycoproteins that prevent the attachment of potential pathogens. Klaus et al., 2020 [64], showed that *Burkholderia thailandensis,* a soil bacterium, produces antimicrobial compounds to inhibit *Bacillus subtilis* colonization in co-culture experiments.

#### 2.2.1. Antifouling Compounds Isolated from Microorganisms

**Figure 2:**
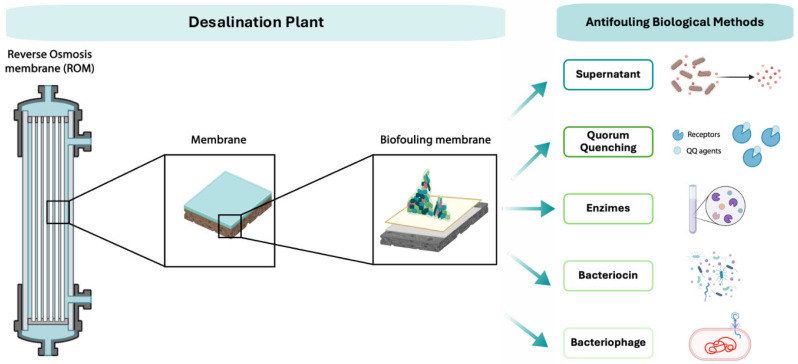
Example of antifouling biological methods.

The antifouling activity originating from biological resources, including bacteria, can be ascribed to various molecules secreted into the extracellular environment, which affect the EPS matrix or modulate bacterial survival. Biological antifouling agents have been identified through investigations in living organisms that demonstrate their efficacy against membrane fouling. These agents exhibit diverse mechanisms of action, application methods, and sources (Figure 2, Table 2).

Cis-2-decenoic acid, a molecule produced by *Pseudomonas aeruginosa*, has been identified as an unsaturated fatty acid capable of inducing the dispersion of biofilms formed by both Gram-positive and Gram-negative bacteria [65]. Additionally, this compound exhibits antifungal activity against yeasts. Conversely, antimicrobial peptides (AMPs) have been synthesized specifically for membrane treatment. These peptides have demonstrated effective antibacterial activity against a broad spectrum of bacteria, thus successfully eliminating biofouling [60,61,62,63,64,65,66]. As a biofilm dispersal signal, controlled release of nitric oxide (e.g., DETA NONOate) significantly mitigated RO biofouling and altered the bacterial community structure [39].

The utilization of enzymes presents a promising approach to address contemporary fouling challenges. In particular, lipases and proteases produced by species such as *Candida cylindracea*, *Pseudomonas mendocina*, *Aspergillus oryzae*, and *Bacillus licheniformis* have shown notable efficacy. The combined use of these enzymes significantly enhances their effectiveness, functioning as industrial catalysts that facilitate the removal of fouling from membranes, thereby promoting the recovery of pure water flow [50]. Conversely, five compounds (phenazine-1-carboxylic acid, 2-n-hyptyl quinol-4-one, 2-n-nonylquinol-4-one, 1-hydroxyphenazine, and pyolipic acid) exhibited antifouling activity against various seawater species and were isolated from the bacterium *Pseudomonas* sp., strain NUDMB50-11 [67].

The issue of fouling is not restricted to membranes but also impacts submerged surfaces. Consequently, various alternatives have been investigated to mitigate this phenomenon. A notable example is portoamides, a cyclic peptide derived from the cyanobacterium *Phormidium* sp. [68]. These proteins have been incorporated into antifouling paint, aiming to inhibit the settlement of mollusc larvae and modify biofilms formed by marine biofouling bacteria [61]. Furthermore, microorganisms such as the actinomycetes *Streptomyces aculeolatus* PTM-029 and PTM-420 produce the terpenoid napyradiomycins, recognized for their antifouling properties. This compound has demonstrated efficacy in preventing the settlement and adhesion of micro and macro species on submerged surfaces [69].

A recent study conducted by Zahidullah et al. (2022) has examined the antimicrobial efficacy of rhamnolipids in the management of biofilm formation and dispersal, as well as their extracellular polymeric substances (EPSs), in reverse osmosis membranes [70,71,72]. The rhamnolipids possess comparative advantages over synthetic chemicals in terms of stability, cost-effectiveness, and reduced toxicity [42,70,71]. Biosurfactants are predominantly employed as cleaning agents due to their properties as surface-active agents that facilitate the solubilization or removal of microbial biomass adhered to surfaces [43,73]. The surfactants most extensively investigated in ultrafiltration and reverse osmosis membranes are those derived from the bacterium *Pseudomonas aeruginosa*, which are produced through biological processes. Notably, rhamnolipids have also been used as efficient UF cleaning agents for whey-fouled membranes, achieving 100% flux recovery under optimized conditions [74]. Additionally, crude ethyl-acetate extracts from the marine bacterium *Pseudomonas aeruginosa* strain RLimb have shown strong antibacterial and antibiofilm activities in lab and panel assays, supporting their potential as eco-friendly antifouling agents [75].

**Table 2:** Presents a summary of key bioactive compounds derived from microbial sources that exhibit antifouling potential, outlining their microbial origin, mechanism of action, and principal targets in membrane biofouling systems.

Compound/Molecule	Microbial Source	Mechanism of Action	Application Target	Refs.
Rhamnolipids	*Pseudomonas aeruginosa*	Disrupt EPS and biofilm matrix	RO membranes	[70,71,72]
Cis-2-decenoic acid	*Pseudomonas aeruginosa*	Biofilm dispersal	Gram +/− bacteria	[65]
AMPs (e.g., HHC-36)	Synthetic/Bacterial	Membrane disruption	Broad-spectrum bacteria	[66]
Lipases/Proteases	*Candida*, *Bacillus* spp.	EPS degradation	Biofilm matrix	[50]
Portoamides	*Phormidium* sp.	Settlement inhibition	Marine biofoulers	[68,76]
Napyradiomycins	*Streptomyces aculeolatus*	Inhibited the bacterial growth	Marine bacterial species	[69]
Lactonases/acilase AHL	*Erythrobacter*, *Labrenzia*, *Pseudomonas* sp., *Bacterioplanes*	Hydrolysis of AHL	RO membranes	[44,58,70,77]
AiiA_S1–5_ and Est_S1–5_	*Pseudoalteromonas* sp. L11 and *Altererythrobacter*	Hydrolysis of AHL molecules	RO membranes	[47]
Nukacin ISK-1	*Staphylococcus warneri* ISK-1	Bactericidal activity	*Staphylococcus aureus* (MRSA)	[78]
Lacticin Q	*Lactococcus lactis* QU 5	Penetrated the biofilm matrix	*Staphylococcus aureus* (MRSA)	[79]
Sakacin 1	*Lactobacillus sakei 1*	Inhibit the early stages of adherence	*Listeria monocytogenes*	[80]
Lactic acid and nisin A	*Lactococcus lactis* UQ2	Suppress growth	*Listeria monocytogenes*	[79]
Colicin	*Citrobacter freundii*	Alter the cell membrane	Multidrug-resistant Gram-negative	[81]
Supernatant	*Alteromonas* Ni1-LEM	Antibacterial activity	RO membranes	[52]
Supernatant	*Vibrio neptunius ULV11*	Antibacterial activity	RO membranes	[51]
(+) Terrein	*Aspergillus terreus* HT5	Permeabilizes membranes using hydroxyl groups	RO membranes	[57]
Bacterial extract	*Pseudomonas aeruginosa Rlimb*	Inhibits initial adhesion and biofilm formation	Marine biofoulers	[75]
Bacteriocins	*Marine bacteria*	AF activity when incorporated into epoxy paint	Marine biofoulers	[62]

#### 2.2.2. Quorum-Quenching

One strategy to mitigate fouling involves the application of quorum-quenching (QQ), a method predicated on the disruption of quorum sensing (QS) [46,82]. QS serves as a cellular communication mechanism that regulates population density through the utilization of signaling molecules, which can be categorized into three principal types: N-acylhomoserine lactones (AHLs), autoinducing peptides (AIPs), and autoinducer-2 (AI-2). These molecules participate in various responses, including biofilm formation and extracellular polymeric substance (EPS) production. QQ molecules are characterized by their capacity to disrupt and inhibit QS-mediated communication among bacteria, thereby obstructing biofilm formation. These molecules may vary in nature (including enzymes, synthetic or natural compounds, among others), mechanisms of action, and biological targets [46,47,77,83].

Three bacterial genera, *Erythrobacter*, *Labrenzia*, and *Bacterioplane*s, have been identified as producing enzymes with potential quorum-quenching (QQ) activity, attributable to their ability to degrade AHL molecules. These enzymes encompass AHL-lactonases, which are capable of degrading the lactone ring; AHL-acylases, which act on the acyl side chain; and AHL-oxidoreductases, which catalyze the reduction of the acyl side chain. The activity of these enzymes leads to the hydrolysis of AHL molecules, resulting in the complete loss of their signaling capability and thus interfering with bacterial communication. Furthermore, two newly identified bacteria belonging to the genus *Pseudomonas* (KS2 and KS10) exhibited endogenous QQ activity [44]. Strain KS2 synthesizes lactonase, while strain KS10 produces acylase. Both strains effectively inhibited biofouling formation in both aerobic and anaerobic microorganisms. Additionally, xanthine oxidase is pertinent to biofouling control, as it catalyzes the oxidation of xanthine to generate hydrogen peroxide and oxygen. These reactive oxygen species are released as byproducts known to disrupt autoinducer-2 (AI-2) signaling and, thus, bacterial communication [84]. Moreover, *Pseudoalteromona*s sp. L11 and *Altererythrobacter* sp. S1–5 also demonstrate potential QQ activity, with four coding genes associated with QQ activity identified in these isolates, including the novel enzymes AiiA_S1–5_ and Est_S1–5_, which target a broad spectrum of AHL molecules [47].

Engineered strategies for quorum-quenching (QQ) have been developed utilizing various methods to immobilize enzymes or bacteria possessing QQ activity. These methods include the application of magnetic carriers for the adherence of QQ enzymes, as well as the use of alginate beads or membranes as matrices for immobilizing enzymes or microorganisms [58,59,60,61,62,63,64,65,66,67,68,69,70,71,72,73,74,75,76,77,78,79,80,81,82,83,84,85]. Two quorum-sensing inhibitor (QSI) application routes are compared—direct dosing into the feed versus covalent/surface immobilization on RO elements—with distinct performance and durability trade-offs [55]. An illustrative example is *Brucella* sp. ZJ1; when encapsulated in alginate beads, it effectively reduced exopolysaccharide (EPS) production and mitigated membrane contamination [45]. *Rhodococcus* sp. BH4 exemplifies another encapsulated bacterium that functions as a QS quenching agent and demonstrates efficacy by enhancing biofouling propensity. Beyond cell or enzyme immobilization, covalent functionalization of the polyamide layer with amphiphilic QSI moieties (via glutaraldehyde-mediated Schiff-base chemistry) has achieved stable biofouling mitigation under real seawater in SWRO modules [56]. Additionally, *Pseudomonas* sp. 1A1, another encapsulated bacterium, exhibited a high rate of acyl homoserine lactone (AHL) degradation, which effectively inhibited biofouling in membranes [86]. Furthermore, a hydrophilic compound secreted by *Acinetobacter* sp. DKY-1 was isolated in 2018, demonstrating the capacity to inactivate the autoinducer-2 (AI-2) signaling molecule [87]. This compound has emerged as a promising antifouling agent. *Microbacterium* sp. has been encapsulated in alginate beads owing to its quorum-quenching (QQ) activity, which degrades acyl-homoserine lactones (AHLs), as validated under anaerobic conditions in AnMBR [88], produced by extracellular polymeric substance (EPS)-producing bacteria. This process reduces membrane biofouling and enhances their efficacy by a factor of eight to ten [81]. Species such as *Kluyvera citrophila*, which secretes penicillin G acylase, have been identified as possessing QQ effects. Its notable characteristic is its high resistance to variations in pH and temperature. This organism directly facilitates the degradation of medium-chain AHLs through the deamidation of these quorum-sensing (QS) signaling molecules [89].

For a method to effectively mitigate fouling formation, particularly through the QQ technique, it is imperative to consider the principal genes affected, which include CylA/CqsA/CqsS/TtrS/TtrR/LuxQ/LuxP/LuxN, in conjunction with genes associated with EPS formation, namely galE/nagB/ilvE/metH/phhA/serB. Inhibition of these genes will result in the blockade of QS signal perception [58].

The Quorum Peps database (https://quorumpeps.ugent.be/ (accessed on 1 August 2025) has been developed to provide a structured description of quorum-sensing (QS) peptides, offering detailed information regarding their origin, functionality, peptide bonds, and chemical characteristics of QS-derived signaling peptides [90].

#### 2.2.3. Bacteriocin

Bacteriocins are antimicrobial peptides derived from bacteria, exhibiting considerable potential due to their diverse functions, which include food preservation, cancer therapies, and treatments against pathogens [60,61,62,91]. Notably, bacteriocins such as nukacin ISK-1, produced by *Staphylococcus warneri* ISK-1, and lacticin Q, extracted from *Lactococcus lactis* QU 5 culture supernatant, display antifouling effects against methicillin-resistant *Staphylococcus aureus* (MRSA). Their significance lies in their capacity to compete directly with antibiotics while also demonstrating efficacy against planktonic cells [60]. Lacticin Q has proven effective against both planktonic cells and biofilms, whereas nukacin ISK-1 exhibits a bacteriostatic effect solely on planktonic cells [78]. *Lactobacillus sakei* 1, a producer of sakacin 1, and *Lactococcus lactis* UQ2, which produces lactic acid and nisin A, exert inhibitory effects on *Listeria monocytogenes* by suppressing its growth and hindering the initial stages of adherence to abiotic surfaces [79,80]. The bacterium *Citrobacter freundii* has been examined, leading to the identification of an antimicrobial compound known as colicin, which demonstrates efficacy against multidrug-resistant Gram-negative bacteria, including *E. coli*, *Citrobacter* spp., and *Klebsiella pneumoniae*, as well as biofilms [81].

#### 2.2.4. Bacteriophage

Bacteriophages represent a viable strategy for addressing biofouling due to their capacity for rapid and efficient infection of the host, which induces the production of enzymes such as depolymerases that degrade the protective matrix (extracellular polymeric substances), thereby facilitating infection [92]. Furthermore, genetically modified phages have been developed to produce enzymes that enhance their effectiveness in biofilm elimination. For instance, the dispersal gene (dspB) was cloned into a phage specific for *Escherichia coli* (T7), resulting in a modified enzymatic phage that demonstrated superior efficacy in biofilm eradication compared to an unmodified phage [48].

### 2.3. Biological Methods Employed Against Membranes in Desalination Systems

Chemical clean-in-place (CIP) protocols based on hypochlorite or strong mineral acids remain the industrial default for mitigating biofouling in spiral-wound reverse osmosis (RO) membranes; nevertheless, the repeated oxidative and hydrolytic shocks inflicted by these reagents accelerate polymer chain scission, shorten module lifetime, inflate operating expenditures, and discharge chlorinated by-products that demand secondary treatment [93,94]. These constraints, together with increasingly restrictive environmental regulations and ambitious corporate sustainability targets, have intensified the search for mechanism-driven, biologically derived alternatives that can be seamlessly integrated into existing desalination workflows without compromising permeate quality. Analogously, full-scale in situ modification of RO elements using glycidyl methacrylate (GMA) has been demonstrated, highlighting permeability–selectivity trade-offs and practical feasibility under plant conditions [95]. Key performance indicators contrasting these biological strategies with benchmark chemical CIP are collated in Table 3.

Unless otherwise stated, changes are relative to the baseline noted in each row (e.g., chemical CIP or clean-membrane control).Marine isolates such as *Alteromonas* sp. Ni1-LEM, *Vibrio neptunius* ULV11, coral- and macroalgae-associated consortia, and *Pseudomonas aeruginosa* secrete cell-free supernatants (CFS) enriched in proteases, glycosidases, DNases, and low-molecular-weight antimicrobial metabolites that either depolymerize extracellular polymeric substances (EPSs) or suppress the metabolic activity of pioneer colonizers [51,52,53,54,103,104]. Polysaccharide lyases (e.g., alginate lyases) degrade biofilm EPS and can potentiate antimicrobials [49]. When evaluated under pilot-scale conditions that replicated industrial pressure, cross-flow velocity, and permeate flux, CFS from *Alteromonas* Ni1-LEM and *V. neptunius* ULV11 restored permeate recovery and membrane topography to levels statistically indistinguishable from conventional CIP, underscoring their potential for large-scale deployment while eliminating the need for post-cleaning neutralization. Representative laboratory, pilot, or full-scale studies are summarized in Table 4.

Biological mitigation extends beyond soluble exudates. The fungal secondary metabolite (+)-terrein isolated from *Aspergillus terreus* HT5 (OP185256) has been covalently grafted onto thin-film composite polyamide membranes via glutaraldehyde chemistry, imparting durable hydrophilicity, broad-spectrum antibacterial activity, and a measurable 8–12% increase in pure-water permeability while simultaneously inhibiting microbial adhesion and irreversible fouling-layer development [57]. Complementarily, in situ post-modification of the active layer with poly(glycidyl methacrylate) has achieved up to a 40% enhancement in the rejection of bisphenol A, carbamazepine, and boric acid without compromising desalination throughput, illustrating how bio-inspired chemistries can be paired with precision polymer engineering to deliver multifunctional membranes [95].

Because N-acyl-homoserine lactones (AHLs) orchestrate microbial surface sensing, attachment, and maturation, disrupting these chemical dialogs offers a highly selective mechanism to curb fouling [90]. Tabraiz et al. quantified ten environmentally relevant AHLs by UPLC-MS/MS in anaerobic membrane bioreactors, providing a performance baseline for quorum-quenching (QQ) efficacy benchmarking [109]. Encapsulating *Rhodococcus* sp. BH4 in poly(vinyl alcohol) beads has been used to degrade AHLs and attenuate fouling in MBR/RO contexts [58,85]. By contrast, Wood et al. engineered a LasI/LasR-regulated, self-controlled *Escherichia coli* ‘beneficial biofilm’ that upregulated the dispersal protein BdcA to self-limit biomass and, with nitric-oxide production, reduced colonization by *Pseudomonas aeruginosa* while maintaining higher normalized flux under cross-flow [110]. Recent reviews of submerged MBRs conclude that quorum-quenching provides a sustainable alternative to intensive hypochlorite dosing for fouling control [58,111,112]. Despite robust bench-scale and pilot-scale evidence, full-scale validation of biological cleaning remains fragmentary. Key hurdles encompass the economic optimization of exudate fermentation and downstream purification, the long-term physicochemical stability of grafted metabolites under high-salinity shear and variable pH, and the regulatory acceptance of live or genetically modified QQ consortia within potable water treatment frameworks [31,32,33,34]. Addressing these gaps through integrated techno-economic analysis, life-cycle assessment, and multi-site field trials will determine whether biological antifouling strategies can transition from promising innovation to a widely adopted industrial standard. The technology readiness level (TRL) of the principal biological approaches is detailed in Table 5.

Collectively, bacterial supernatants, metabolite-functionalized membranes, and QS-targeted interventions constitute a convergent, modular toolkit capable of mitigating biofilm formation, preserving membrane integrity, and enhancing trace-contaminant rejection—thereby aligning with both the operational imperatives and the environmental stewardship goals of next-generation desalination infrastructures.

## 3. Challenges and Future Perspectives

Although the suite of biotechnological innovations reviewed herein represents a promising and inherently sustainable toolkit for mitigating biofouling in pilot-scale desalination facilities, a series of interrelated scientific, engineering, and regulatory challenges must still be overcome before broad industrial deployment can be contemplated. The most pressing knowledge gaps and development bottlenecks are summarized below. Long-term stability data remain sparse in field settings.

a. Technoeconomic scalability: To make biological alternatives for biofouling control competitive in industrial-scale reverse osmosis (RO), the primary constraints are scale and cost rather than the agent’s intrinsic potency. At plant scale, an anti-biofouling supernatant from *Alteromonas* sp. Ni1-LEM was produced and validated at 400 L. The working solution (200 µg mL^−1^) cost USD 0.14–0.19 L^−1^ and achieved higher permeability recovery than the reference chemical protocol (PR ≈ 0.7205 vs 0.6873), while restoring RO-membrane (ROM) topography without compromising integrity [52]. Consequently, techno-economic targets should be framed as the cleaning volume required per square meter of membrane for each plant, rather than as a single USD-per-m^2^ figure. To meet these cost targets, the available evidence supports strain and fermentation engineering/optimization, use of low-cost substrates, and intensified downstream processing (e.g., ultrafiltration, adsorption, foam fractionation) to reduce solvent and energy use, given that downstream processing can account for ≈ 60% of total cost [43,70].

b. Physicochemical robustness and controlled delivery: Candidate molecules must retain bioactivity under the combined stresses of hypersalinity (>70 g L^−1^ NaCl), elevated transmembrane pressures (up to 70 bar), and wide pH excursions encountered during chemical cleaning [113]. Sophisticated micro/nanoencapsulation systems—capable of withstanding shear and gradually releasing actives in response to local quorum-sensing signals—remain an outstanding material science challenge that will dictate field longevity.

c. Environmental fate and regulatory acceptance: Despite their inherent biodegradability, the chronic ecotoxicity of transformation products and their propensity to accumulate in brine streams are poorly characterized. Life-cycle assessment coupled with probabilistic environmental risk assessment, aligned with the EU Water Framework Directive and USEPA guidelines, will be indispensable to secure permitting and public acceptance.

d. Hybrid membrane architecture: Rationally designed thin-film nanocomposites that co-immobilize bioactives with metal–organic frameworks or two-dimensional nanomaterials could deliver synergistic antifouling, antiscaling, and high selectivity performance; however, scalable interfacial polymerization routes that preserve both membrane integrity and bioactive functionality are still lacking. Aligned regulatory frameworks include the WHO Guidelines for Drinking-water Quality [31], EFSA QPS [32], US EPA TSCA biotechnology regulations [33], and the EU Water Framework Directive [34]. Accordingly, we prioritize cell-free approaches and immobilized enzymes for potable water contexts.

e. Predictive modeling and digital twins: Multiphysics simulations that combine computational fluid dynamics with molecular-scale interaction models are urgently needed to forecast the spatiotemporal distribution of bioactives, biofilm evolution, and resultant hydraulic impacts under dynamic operating scenarios [19].

Consistent with Baransi-Karkaby et al. (2019) [95], the ~20% permeability loss reported in our trials was outweighed by a marked suppression of concentration polarization, translating into superior permeate quality and reduced specific energy demand. Continued advances in synthetic biology, encapsulation technology, and in situ monitoring are, therefore, poised to enable seamless integration of these biobased strategies into full-scale reverse osmosis and ultrafiltration trains, ultimately enhancing the circularity, energy efficiency, and resilience of desalination and allied water treatment sectors.

## 4. Conclusions

Biofouling remains a critical bottleneck in industrial water treatment infrastructure, especially at reverse osmosis desalination plants. Conventional countermeasures—pretreatment trains, surface coatings, and periodic physical–chemical cleanings—have delivered only incremental gains, often below the reliability and sustainability thresholds required by next-generation facilities. During the past decade, however, biologically inspired approaches centered on enzymatic hydrolases, quorum-quenching proteins, biosurfactants, and other bacterially derived metabolites have opened an evidence-based path toward substantive fouling control.

Deploying these bioactive agents as in situ cleaning or conditioning additives reframes fouling mitigation as an ecological process that harnesses, rather than resists, microbial activity. Realizing this vision demands concerted progress in strain engineering, fermentation scale-up, formulation chemistry, and delivery hardware, together with transparent regulatory frameworks and proactive engagement from plant operators, equipment vendors, and environmental authorities.

Convergent advances in synthetic biology, nanoconjugation, and data-driven process engineering are poised to overcome present performance and cost constraints, positioning customized bioactive cocktails as routine elements of desalination plant operating procedures. To accelerate adoption, future research must couple pilot-scale demonstrations with rigorous life-cycle and techno-economic assessments, address ecological trade-offs through mesocosm-based risk analysis, and establish harmonized guidelines for efficacy testing and discharge compliance. By integrating these scientific, engineering, and policy dimensions, the field can deliver fouling-resilient desalination that maximizes water-production efficiency while minimizing environmental footprint, thereby advancing the broader agenda of sustainable resource management.

## Figures and Tables

**Table 1:** Advantages and disadvantages of various antifouling methods employed in reverse osmosis membranes.

		Advantage	Disadvantage	Refs.
Antifouling methods	** Chemical **	High effectiveness in mitigating fouling.	Significantly toxic to non-target organisms.	[26,28,30,43]
	** Physical **	Efficient in controlling fouling, enhancing water flow.	Potential for long-term damage to membranes, coupled with considerable costs.	[1,31,32,33,34,44,45]
	** Biological **	High specificity in the regulation of fouling.	Insufficiently researched, necessitating implementation on a pilot scale.	[36,41,46,47,48]

**Table 3:** Comparative evaluation of biological vs. chemical treatments to mitigate biofouling on reverse osmosis (RO) membranes.

Approach (Type)	Mechanism/Typical Agent (s)	Target Biofilm Stage	Key Benefits	Limits/Risks	Performance (Verified Example)	Best-Use Cases	Refs
*Alteromonas* sp. Ni1-LEM supernatant (biological cleaner)	Peptide/enzymatic CFS (~200 μg·mL^−1^) disrupts EPS; ambient cycles.	Mature EPS-rich biofilm on PA SWRO (3.5-yr fouled ROMs).	Higher Pi/PR and lower conductivity vs. chemical; AFM recovery.	Batch-to-batch QC; scale-up and SOP standardization.	Pi 0.3747 vs. 0.3625; PR 0.7205 vs. 0.6873; lowest conductivity.	Green CIP/hybrid when EPS dominates; avoids high-pH on PA.	[52]
*Vibrio neptunius* ULV11 CFS (biological cleaner)	CFS (~250 μg·mL^−1^) anti-EPS + antibacterial.	Fouled ROMs; operational CF042 at high pressure/flux.	Flux/permeate ≈ chemical; conductivity < 600 vs. ~615 μS·cm^−1^.	Composition characterization ongoing; manufacturing/QA.	Flux/permeate comparable; CLSM (ConA) ↓; AFM/SEM restored.	Biological CIP in plant; removes EPS + biomass.	[51]
Xanthine oxidase + hypoxanthine (enzymatic, ROS-assisted)	XO + hypoxanthine → ROS degrades polysaccharides.	Mature EPS on industrially fouled PA RO membranes (bench cross-flow).	Near-clean flux; ~50% polysaccharides ↓; rejection preserved.	Dose control; ROS management; needs pilot-scale delivery to spiral-wound modules.	Flux ~86.7% of clean; polysaccharides ↓ ~50%.	Targeted EPS removal; pre-rinse.	[84]
Quorum-quenching (QQ) bacteria/enzymes (preventive)	QQ degrades AHL; immobilized columns.	Early/mid-stage formation (signal disruption).	Non-toxic; continuous use.	Lab/pilot; immobilization/regulatory. In MBRs, exogenous QQ may shift communities and briefly raise biofouling—place carefully.	Lab-RO: less biofouling; AHL degraded.	Pretreatment loops to delay refouling.	[85,96]
Enzymatic CIP (lipase/protease)	Protease/lipase blends (± surfactant) solubilize organics/EPS.	Mature organic/EPS foulants on PA TFC membranes.	Similarly to strong caustic; less refouling; keeps hydrophobicity.	Cost/compatibility; recipe/time optimization.	Multi-cycle RO effective; abattoir UF: ≈ 100% PWF; lipids/proteins ↓.	Minimize caustic; sensitive PA.	[50,97,98]
Conventional chemical CIP (alkaline/acid)	STPP/EDTA pH ≈ 12, 35 °C + 1% citric; optional oxidants.	Mixed foulants (organics, biofilm, colloids, inorganics).	Standard; broad efficacy.	PA oxidation; higher chem/energy; sometimes worse than EPS-targeted bio-cleaners.	CF042 PR 0.6873 vs. Ni1-LEM 0.7205; higher conductivity. Wastewater RO: NaOH–SDS → HCl; 35 °C/25 min; cross-flow 0.5 → 1.5 m·s^−1^ → FR 38%→90%.	Routine CIP; mixed foulants; post-algal.	[13,52,99]
Pretreatment + non-oxidizing biocides (chemical)	DAF/DMF + LAE, DBNPA, MIT (where allowed).	Upstream biomass/TOC/AOC control; prevention.	Lowers RO load; avoids PA oxidation.	Regulatory/health; weak on mature biofilms.	Full-scale DMF: low removal with frequent chlorination; LAE strong anti-biofilm (PA coupons).	Integrated pretreatment; periodic dosing.	[100,101]
Chlorination (chemical, upstream)	NaOCl upstream; dechlorinate before PA RO.	Upstream bioburden control.	Simple; common (CTA).	PA oxidation risk; adaptation; may worsen biofouling; neutralize fully.	Mixed; PA damage risk; CTA more tolerant.	CTA; intake shock dosing with full dechlorination.	[99]
Biosurfactant cleaners (rhamnolipids)	Lower surface tension; detach proteins/EPS; ↑ permeability.	Mature (protein/EPS layer) and early stage (initial adhesion).	Biodegradable; low-dose efficacy (~0.3 g·L^−1^).	Cost/availability; foaming; limited RO data; verify PA compatibility.	Whey UF: FR 100% @ 0.3 g·L^−1^ (SDS 84%; ~NaOH 4 g·L^−1^); dead-end: +20% flux @ 6 h/300 μg·mL^−1^; no damage after 4 cycles.	Gentle CIP; frequent/inter-cycle; co-cleaner alternative.	[74,102]

Abbreviations: RO = reverse osmosis; ROM = reverse osmosis membrane; PA = polyamide; EPS = extracellular polymeric substances; CFS = cell-free supernatant; Pi = water permeability; PR = permeability recovery; CLSM = confocal microscopy; AFM = atomic force microscopy; DAF/DMF = dissolved air flotation/dual-media filtration; LAE = lauryl arginate ethyl. Symbols: ↑: increase; ↓: decrease; →: “to/then/produces” (indicates progression or change from the left value/condition to the right).

**Table 4:** Selected laboratory, pilot, and full-scale demonstrations of biological fouling control.

Plant/Location	Flow	Membrane Type	Biological Agent	Dose or Mode	Flux Gain	TPM	Chemical Cleaning Cycles (CIP) Replaced	Ref.
Aguas Antofagasta SWRO (N. Chile)	1056 L/s (installed)	SWC6-LD spiral-wound PA (Hydranautics)	*Alteromonas* sp. Ni1-LEM supernatant	Working soln 200 µg/mL (total protein); substituted acid/alkaline CIP; tests @58 psi (4 bar)	NR (PR recovery reported)	58 psi (~4 bar) in cleaning protocol	Replaced: 1% citric ± 0.1% EDTA pH 12 (Sterlitech CF042)	[52]
Aguas Antofagasta SWRO (N. Chile)	1056 L/s (installed)	SWC6-LD spiral-wound PA (Hydranautics)	*Vibrio neptunius* ULV11 supernatant	Working soln 100 µg/mL (total protein); substituted acid/alkaline CIP; tests @58 psi (4 bar)	NR (PR recovery reported)	58 psi (~4 bar) in cleaning protocol	Replaced: 1% citric ± 0.1% EDTA pH 12 (Sterlitech CF042)	[51]
RO plant (Mansehra, Pakistan); lab tests	NR	RO, TW30-1812-100HR (DOW)	Rhamnolipids (biosurfactants)	100–1000 mg/L; 2 h dispersal (viability and EPS-reduction assays)	NR	NR	NR	[71]
Lab RO (NTU, Singapore)	NR	RO (unspecified)	Recombinant quorum-quenching (QQ) bacterium	Direct dosing or immobilized in a microfilter to degrade AHLs	NR	NR	NR	[83]
Two full-scale SWRO plants (DAF–UF vs. DMF–CF)	NR	SWRO (full-scale)	NR (BGP method study via flow cytometry)	NR	NR	NR	Compared the CIP frequency between DAF–UF and DMF–CF (no replacement)	[105]
NR (review)	NR	Dominant commercial RO: fully aromatic composite PA (spiral-wound)	NR	NR	NR	NR	NR	[106]
Lab tests (University of Copenhagen et al.)	NR	RO (PA) coupons	Enzymes (Trypsin-EDTA, Proteinase K, α-Amylase, β-Mannosidase, Alginate lyase)	0.05–1.28 U/mL; Proteinase K 100 µg/mL; Trypsin-EDTA 0.0125%; mixes for 4–24 h	NR (biovolume reduced 43–71%)	NR	NR	[107]
Full-scale SWRO plants (Middle East and Australia case studies)	NR	SWRO (full-scale)	NR (ATP/BGP methods used for monitoring)	NR	NR	NR	NR	[108]

Abbreviations: NR = not reported; RO = reverse osmosis; SWRO = seawater reverse osmosis; PA = polyamide; CIP = Clean-in-Place; AHL = N-acyl-homoserine lactone; ATP = adenosine triphosphate; BGP = bacterial growth potential; TPM = value as reported by authors (if not reported, shown as NR).

**Table 5:** Technology readiness level (TRL) of biological antifouling solutions.

Solution Class/Agent	Scale and System Evaluated	Quantitative Result Reported	Suggested TRL (2025; Based on Cited Evidence)	Costs Reported in the Paper?	Refs.
Enzymatic cleaners (protease + lipase)	Bench UF cleaning of abattoir-fouled membranes; blend vs. single enzymes; surfactant compatibility.	FRR up to ~98.4% with *Pseudomonas* lipase + Protease A; blend > single enzymes.	3–4 (lab; no pilot).	NR.	[50]
Biosurfactant cleaning (rhamnolipids)	Bench RO and cellulose acetate; dead-end and cross-flow.	~20% flux rise at 300 µg mL^−1^ for 6 h (dead-end); under cross-flow outperformed a commercial chemical cleaner.	3–4 (bench).	NR.	[102]
Quorum-quenching bacteria (*Rhodococcu*s BH4; PVDF ‘bag’)	Lab MBR (~5 L) with PVDF ‘bag’ containing *Rhodococcus* BH4; tracked TMP, EPS, AHL.	Slower TMP rise; lower TB-EPS; C8-HSL half-life cut from ~4 h to ~1 h.	4 (lab MBR; QQ modality reported up to 6).	NR.	[58,85]
Engineered bacteriophage (T7-DspB)	MBEC biofilm model with *E. coli*; T7 engineered to express dispersin B.	~4.5-log reduction (~99.997%); ~2-log better than control; effective 10^1^–10^5^ PFU; SEM confirms.	2–3 (in vitro).	NR.	[74]
Bacteriocins against MRSA biofilms (nisin A, lacticin Q, nukacin ISK-1)	In vitro staphylococcal biofilms at 4× MIC; compared with vancomycin.	In planktonic MRSA (MR23, 4× MIC), nisin A kills in 1h; lacticin Q in 4h. Biofilms are more tolerant; 4× MIC/24 h: nisin A ~10×; vancomycin ineffective in biofilms.	2–3 (medical in vitro).	NR.	[78]
Enzymatic QQ on magnetic carriers + magneto-filter	Lab MBR with acylase immobilized on magnetic carriers (≈3 mg g^−1^) and external MF.	TMP ≈ 10 kPa for ≈200 h vs. <50 h in control.	4–5 (prototype lab; related field evidence).	NR.	[58]
Bacterial supernatant (*Alteromonas* sp. Ni1LEM)	CF042 RO using SWC6LD ROMs in service ~3.5 y; 825 psi; 15 h supernatant-only cleaning.	Pi = 0.3747 vs. 0.3625 (chemical); PR = 0.7205; permeate 469.5 µS cm^−1^ vs. 490.7; AFM shows restoration.	4 (relevant lab validation).	Yes: ~USD 0.14–0.19 L^−1^ (200 µg mL^−1^ protein).	[52]
Cell-free supernatant (*Vibrio neptunius* ULV11)	CF042 at 825 psi; SWC6LD ROMs used 3–3.5 y; plant-like conditions; AFM/SEM/CLSM.	Flux/permeability comparable to chemical; permeate < 600 µS cm^−1^ vs. ~615; strong EPS reduction (ConA).	4 (relevant lab validation).	NR.	[51]
Enzymatic cleaning (xanthine oxidase + hypoxanthine)	Bench cross-flow (600 psi) with industrially fouled RO ROMs; artificial seawater; 20 h.	Flux ≈ 8.4 L m^−2^ h^−1^ (~86.7% of clean 9.7); ~50% less polysaccharides; minimal biocidal action.	3–4 (bench; relevant conditions).	NR (enzyme high cost; no figures).	[84]

Readiness Levels (TRLs) are suggested from the experimental context reported (lab bench, lab-scale membrane bioreactor, pilot/field) using a 1–9 scale (3 = proof-of-concept, 4 = lab validation in a relevant environment, 5–6 = pilot/field demonstration). The cost column reflects whether the paper itself reported quantitative costs—if not, it is marked as Not Reported (NR).

## Data Availability

No new data were created or analyzed in this study. Data sharing is not applicable to this article.

## Abbreviations

The following abbreviations are used in this manuscript:

AFM	Atomic force microscopy
AHL	N-acyl-homoserine lactones
BODIPY	Boron–dipyrromethene fluorophore (e.g., BODIPY–vancomycin)
BWRO	Brackish water reverse osmosis
CF042	Bench crossflow RO test cell/skid
CFS	Cell-free supernatant
CFS-ULV11	Cell-free supernatant from *Vibrio neptunius* strain ULV11
CFU	Colony-forming units
CIP	Clean-in-place
CLSM	Confocal laser scanning microscopy
ConA	Concanavalin A lectin stain for polysaccharides
CRI	Commercial readiness index
DspB	Dispersin B (PNAG-degrading enzyme)
EPS	Extracellular polymeric substances
EU	European Union
GDWQ	Guidelines for Drinking-water Quality
HSL	Homoserine lactone (e.g., C8-HSL)
MBEC	Microtiter peg biofilm model (Montana Biofilm Device)
MBR	Membrane bioreactor
MIC	Minimum inhibitory concentration
MF	Magneto-filter (magnetic retention unit)
NR	Not reported
QQ	Quorum-quenching
QS	Quorum sensing
QSI	Quorum-sensing inhibitors
PFU	Plaque-forming units
Pi	Water permeance (L·m^−2^·h^−1^·bar^−1^)
PNAG	Poly-N-acetylglucosamine (biofilm exopolysaccharide)
PR	Permeability/flux recovery (dimensionless fraction)
PVDF	Poly(vinylidene fluoride)
RO	Reverse osmosis
ROMs	Reverse osmosis membranes
ROS	Reactive oxygen species
SWRO	Seawater reverse osmosis
SEM	Scanning electron microscopy
SMP	Soluble microbial products
TB-EPS	Tightly bound extracellular polymeric substances
TMP	Transmembrane pressure
TRL	Technology readiness level
TSCA	Toxic Substances Control Act
UF	Ultrafiltration
US EPA	United States Environmental Protection Agency
UVC	Ultraviolet-C
WFD	Water Framework Directive
WHO	World Health Organization
